# Assessing breed integrity of Göttingen Minipigs

**DOI:** 10.1186/s12864-020-6590-4

**Published:** 2020-04-16

**Authors:** Christian Reimer, Ngoc-Thuy Ha, Ahmad Reza Sharifi, Johannes Geibel, Lars Friis Mikkelsen, Martin Schlather, Steffen Weigend, Henner Simianer

**Affiliations:** 1 Department of Animal Sciences, Animal Breeding and Genetics Group, University of Göttingen, Albrecht-Thaer-Weg 3, 37017 Göttingen, Germany; 2 Center for Integrated Breeding Research, University of Göttingen, Albrecht-Thaer-Weg 3, 37075 Göttingen, Germany; 3 Ellegaard Göttingen Minipigs A/S, Soroe Landevej 302, 4261 Dalmose, Denmark; 4 Institute of Mathematics, University of Mannheim, B6 26, 68131 Mannheim, Germany; 5 Institute of Farm Animal Genetics of the Friedrich-Loeffler-Institut, Höltystraße 10, 31535 Neustadt, Germany

**Keywords:** Göttingen Minipigs, Conservation, Population structure

## Abstract

**Background:**

Göttingen Minipigs (GMP) is the smallest commercially available minipig breed under a controlled breeding scheme and is globally bred in five isolated colonies. The genetic isolation harbors the risk of stratification which might compromise the identity of the breed and its usability as an animal model for biomedical and human disease. We conducted whole genome re-sequencing of two DNA-pools per colony to assess genomic differentiation within and between colonies. We added publicly available samples from 13 various pig breeds and discovered overall about 32 M loci, ~ 16 M. thereof variable in GMPs. Individual samples were virtually pooled breed-wise. F_ST_ between virtual and DNA pools, a phylogenetic tree, principal component analysis (PCA) and evaluation of functional SNP classes were conducted. An F-test was performed to reveal significantly differentiated allele frequencies between colonies. Variation within a colony was quantified as expected heterozygosity.

**Results:**

Phylogeny and PCA showed that the GMP is easily discriminable from all other breads, but that there is also differentiation between the GMP colonies. Dependent on the contrast between GMP colonies, 4 to 8% of all loci had significantly different allele frequencies. Functional annotation revealed that functionally non-neutral loci are less prone to differentiation. Annotation of highly differentiated loci revealed a couple of deleterious mutations in genes with putative effects in the GMPs .

**Conclusion:**

Differentiation and annotation results suggest that the underlying mechanisms are rather drift events than directed selection and limited to neutral genome regions. Animal exchange seems not yet necessary. The Relliehausen colony appears to be the genetically most unique GMP sub-population and could be a valuable resource if animal exchange is required to maintain uniformity of the GMP.

## Background

The Göttingen Minipig (GMP) is an animal model with growing importance [1]. Created in the 1960’s by crossing Minnesota Minipigs, Vietnamese Potbellied Pigs and the German Landrace, the breed has been under a fully documented closed breeding scheme ever since. The first colony was founded at the research farm of the University of Göttingen in Friedland, Germany, and later resettled to the Relliehausen research farm. Due to the growing customer interest of using GMPs, this facility could not satisfy the demand anymore and therefor collaboration with Ellegaard Göttingen Minipigs A/S in Dalmose, Denmark, including a larger colony, was established in 1992. In 2003, animals from this colony were brought to Marshall BioResources, North Rose, New York, USA as the basis of a North American GMP breeding colony. In 2009, a second barrier colony was established in Dalmose, based on breeders from the first barrier colony to increase the production. Finally, animals from Dalmose were brought to Oriental Yeast Co., Ltd. in Nisshin, Japan, in 2013 to establish a barrier colony in Japan. After the initial animal transfer, all colonies remained under closed breeding without any genetic exchange, albeit being under a common controlled breeding scheme, coordinated by the animal breeding and genetics group at the University of Göttingen, Germany. Today all GMPs under that management are bred for two main traits, number of piglets born alive and an index comprising body weight measures at different ages. It is inherent in breeding practice that there is also a non-documented co-selection on tame behavior and against appearing malformations such as unwanted pigmentation [2]. Different intensities of conservational breeding techniques, such as adjustment of selective pressures and balancing selective pressures between the sexes, is used to account for the different herd sizes, eventually results in all colonies having comparable effective population sizes.

Managing the GMP in independent colonies in closed barriers is beneficial from a safety point of view. Additionally, a production unit close to the main sales market minimizes negative effects on animal welfare through long transports and prevents import complications and unnecessary bureaucracy. On the other hand, splitting a population reduces the effective population size of each sub-population, which increases the risk of genetic drift or the manifestation of recessive disorders [3]. Two concepts to counter these risks are purging of deleterious variants [4] or maintenance of genetic diversity [5]. Lacy [6] argues that drift is the most important factor in loss of genetic variance when effective population sizes are low, as in the case of the GMP [7], and the only effective measure to mitigate adverse effects would be animal exchange.

In this study we try to assess whether the genetic management was able to maintain the uniformity of the GMP breed, or if the isolated production units are already genetically diversified such that an exchange of breeders is inevitable. This was done by re-sequencing two representative DNA pools from each unit: candidates were sampled for low average relationship within a pool, but elevated relationship towards the remaining colony, allowing an assessment of the diversity within and between units.

## Results

Sampling of the optimally representative candidates for pooling based on relationship measures resulted in candidate sets which exhibited lower inner-set mean relationship coefficients (*a*) compared to the mean relationship of the candidate set with the remaining colony mates (*b*). Both, the absolute level of relationship and the difference between *a* and *b* were lowest for Relliehausen (RE) and highest for North Rose (NR), while Dalmose barrier 2 (DA2) and 3 and Nisshin (NI) were at the same level and exhibited similar difference between a and b (Table 1).

**Table 1:** Mean coefficients of relationship within a colony sample and between the sample and the remaining stock, including number of successfully extracted probes

	RE	DA2	DA3	NR	NI
Relationship within sample	0.357	0.396	0.396	0.403	0.387
Relationship Sample/ Remaining Stock	0.376	0.404	0.402	0.410	0.397
Successful DNA extractions	28	24	23	28	24

Variant calling discovered 67′056’755 raw biallelic SNPs. After the variant quality score recalibration and filtering, 32′615’461 (incl. 945′565 SNPs on chr. X) total non-monomorphic SNPs, thereof 4′121’427 (incl. 263′498 on chr. X) novel SNPs not documented in dbSNP were retained. Discarding loci, monomorphic in the minipigs and X-chromosomal loci for the analysis within minipigs left a data set containing 16′498’773 autosomal SNPs.

### Principal component analysis

**Fig. 1:**
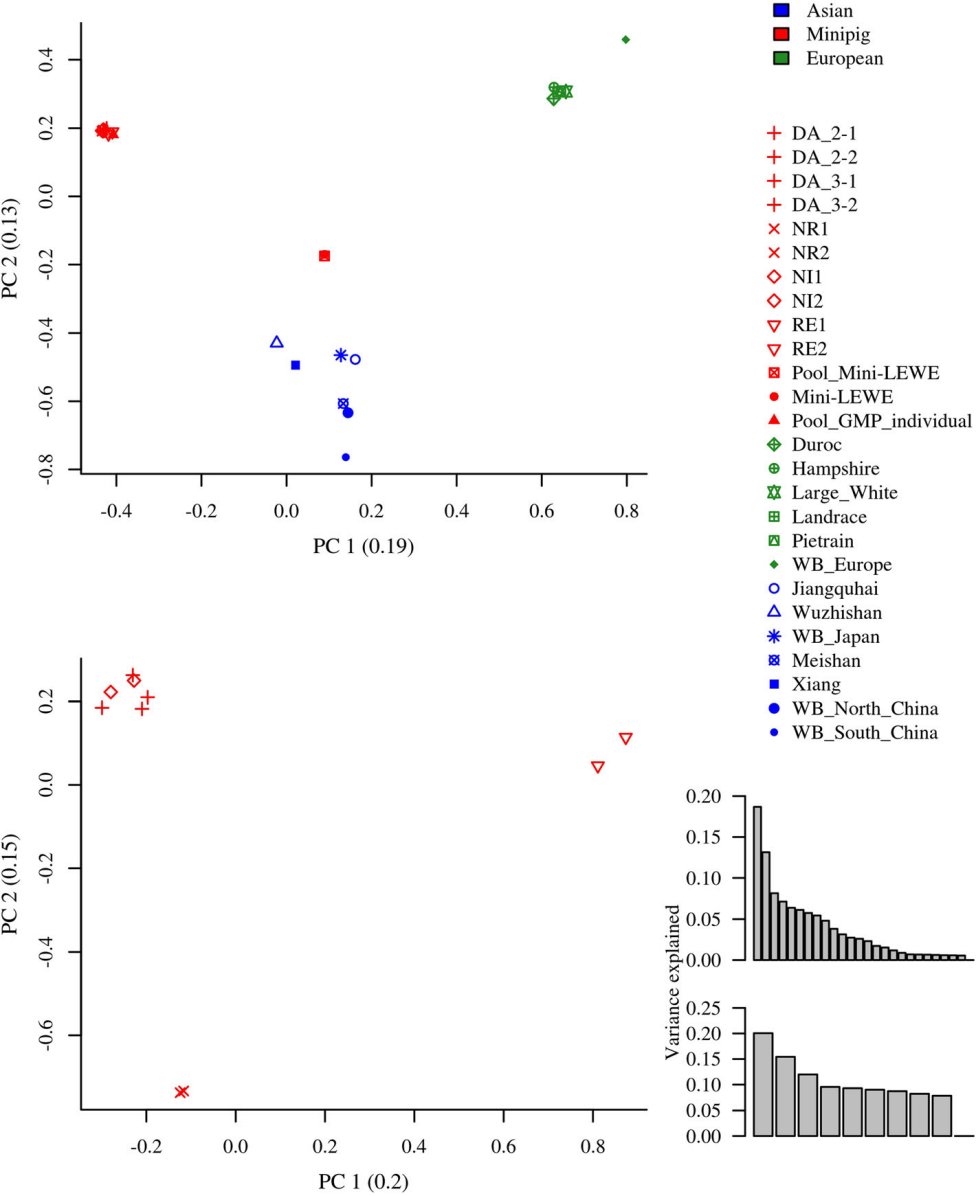
PCA based on pairwise comparisons of reference allele frequencies in all pools (top) and GMP DNA pools only (bottom); Variance explained by PC in brackets. Distribution of variance explained by PC’s on the right

Principal component analysis (PCA) on the reference allele frequencies shows clear separation of the GMPs, Asian and European breeds, with the Mini-LEWE clustering close to the Asian breeds, by the first component (Fig. 1). The second component separates the Asian breeds from GMPs and Europeans. In the context of multiple breeds, no structure can be observed within the GMPs. Separate analysis shows three sub-groups within the GMPs consisting of the RE colony, NR colony and a composite group of DA2, DA3 and NI.

### Differentiation and distance measures

**Fig. 2:**
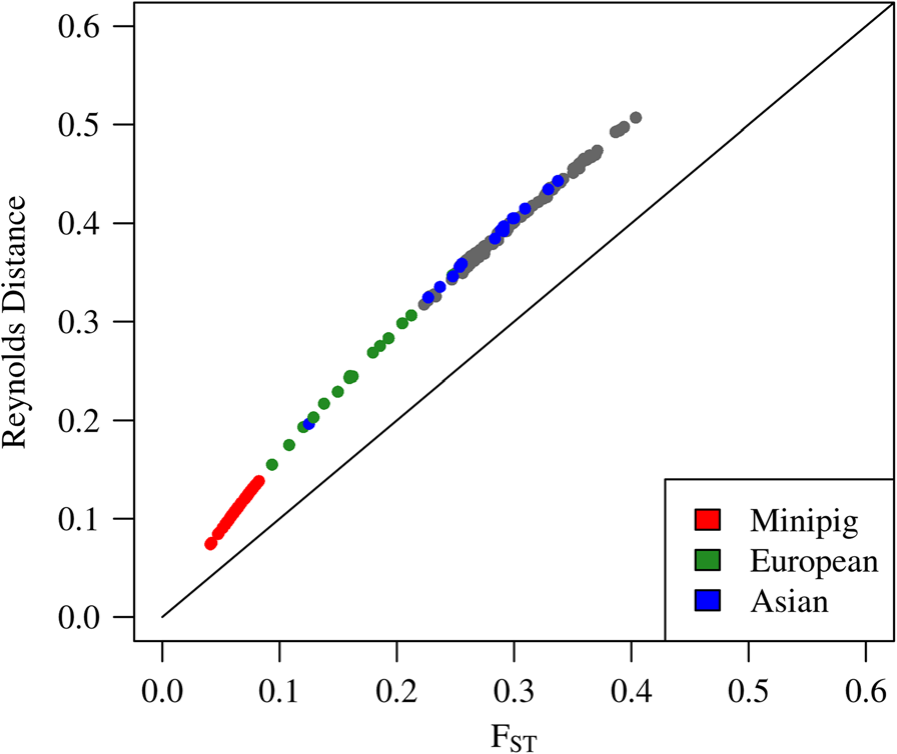
Genome wide FST vs. Reynolds distances for all pairwise comparisons. Comparisons within breed types in the respective colors, comparisons between breed types in grey

As measures of differentiation and genetic distance between the different breeds, F_ST_ and the Reynolds genetic distance (D_R_) were estimated. When applied to the variable set of large breeds and minipig pools, both measures provided a similar picture of three strongly differentiated groups (Table 2). In principle, these three groups were the minipigs, the European breeds and the Asian breeds, respectively, with the exception, that the Mini-LEWE pool clustered with the Asian group. Comparing F_ST_ against D_R_, the latter showed generally higher estimates, relatively inflated at moderate levels of differentiation/ distance (Fig. 2), but provided in general a very similar picture. Therefore, only F_ST_ was used for later purposes, such as the functional annotation. Focusing on the differentiation within the three groups, the GMP exhibited the lowest average differentiation (F_ST_: 0.07; D_R_: 0.11; see also Additional File 1: Supplementary Table 1), the European (F_ST_: 0.16; D_R_: 0.25) the second lowest and the Asian the highest (F_ST_: 0.27; D_R_: 0.37) (Table 2). The average differentiation to other groups was higher than the differentiation within the groups, clearly so for minipigs and for European breeds, but not that clearly for the Asian breeds. The latter exposed an even lower average D_R_ (0.36 vs 0.37) and F_ST_ (0.26 vs. 0.27) to the GMP than within the group of Asian breeds.

**Table 2:** Mean F_ST_ and D_R_ between European and Asian breeds and GMP

		GMP	Asian	European
	GMP	0.066	0.262	0.307
FST	Asian		0.271	0.327
	European			0.163
	GMP	0.113	0.364	0.408
D_R_	Asian		0.372	0.428
	European			0.246

### Phylogeny

**Fig. 3:**
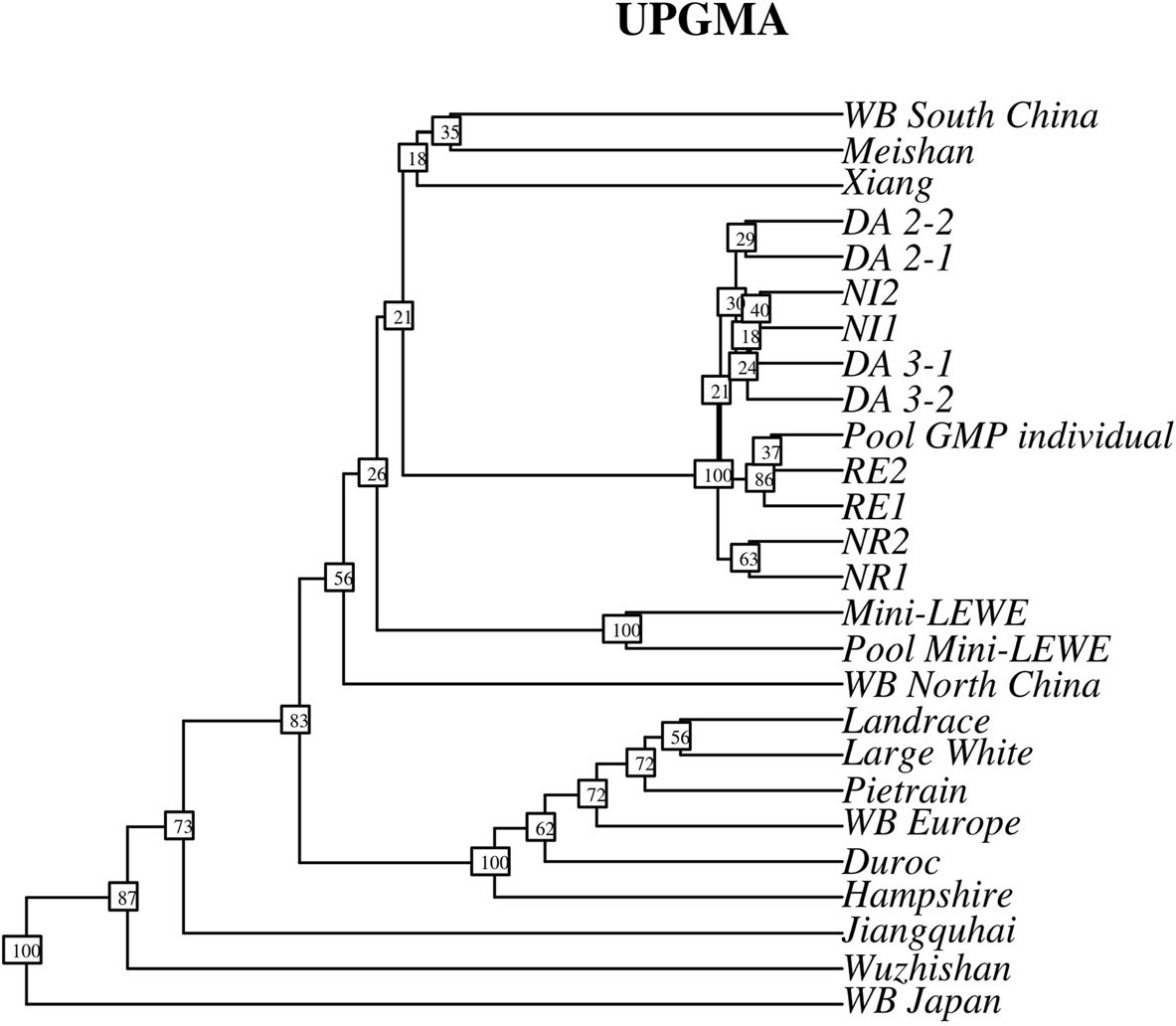
UPGMA tree based on genome-wide FST values; resampling frequency based on 100 random samples of 100 loci in rectangles

The UPGMA tree (Fig. 3) produced from F_ST_ values calculated with genome wide SNP data shows a clear clustering of the GMPs from the other breeds. The next level clusters contain (in that order) Xiang, Meishan, South Chinese wild boars, the Mini-LEWE and the North Chinese wild boars. The European breeds form their own cluster. Throughout 100 resamplings, the GMP cluster, the Mini-LEWE cluster and European cluster are rediscovered in every iteration, while the nodes connected to the Asian breeds seem unstable with resampling probability between 18 and 87%, with the exception of the Japanese wild boar, that behaves like an outgroup sample. Even though, the European and the GMP clusters are distinct, the order within the clusters is variable. The node support within the European cluster spans from 56 to 72%, and between 18 to 86% in the GMP cluster. The most stable structure with 86% contains the RE pools and the least stable (18%) contains the DA and NI herds.

### Stratification within the GMP

The genetic differences within the GMP were determined by comparing pools in terms of allele frequency differences, such as oppositely fixed alleles, extreme F_ST_ values between colonies, differences in the average expected heterozygosity within pools by a variation based approach employing an F-test statistic. Resulting loci detected by the aforementioned statistics were functionally annotated and imbalances between the various classes were checked for potential biases towards differentiated loci.

### Significance test of pool allele frequencies between and within colonies

**Fig. 4:**
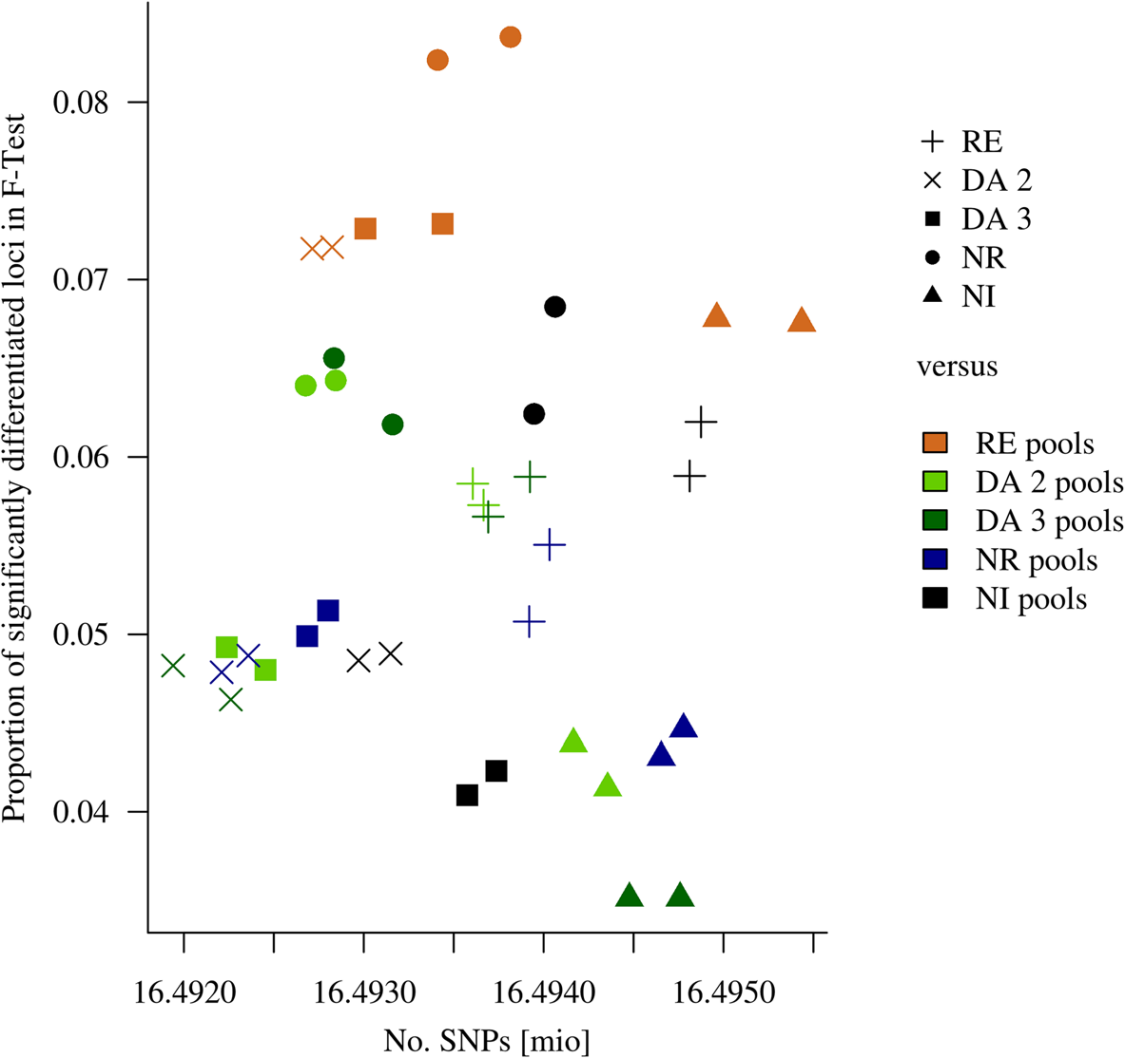
Proportion of significantly different loci at 5% Bonferroni corrected F-test level against number of tested loci

The F-test compared the variation between the two pools with the variation between one of the pools against one foreign pool and could, in contrast to F_ST,_ add probabilistic evidence on differentiation between pools. On average, the NI colony had the lowest proportion of significantly (*p* = 0.05, Bonferroni corrected) differentiated loci, overall 4.7%, followed by DA3 and 2 with 5.3 and 5.4%, respectively, and RE with 5.7%. With 6.9%, NR had the highest proportion of significantly differentiated loci (Table 3; Supplementary Table 2). Focusing on the colonies separately (Fig. 4), only RE had comparable amounts of differentiated loci with all others. From the perspective of DA, NI, and NR, the level of differentiation to RE was clearly highest throughout all comparisons, while the number of evaluated loci was relatively even throughout the comparisons. This means, e.g. RE pools could be significantly different from NR at a locus, since Variation within NR is low, and the average allele frequency in RE is far while using the more variable RE as a basis, the NR frequency is ranging within the variation of RE. Mostly both tested pools of a colony showed a similar amount of differentiation with the exception of NR versus the two NI pools. The highest proportion of differentiated loci was found, when NR was tested against the two RE pools.

**Table 3:** Proportion of SNP significantly different between colony and remote pool in F-test at 5%

	RE_1	RE_2	DA2_1	DA2_2	DA3_1	DA3_2	NR_1	NR_2	NI_1	NI_2
RE	0.00	0.00	0.06	0.06	0.06	0.06	0.06	0.05	0.06	0.06
DA2	0.07	0.07	0.00	0.00	0.05	0.05	0.05	0.05	0.05	0.05
DA3	0.07	0.07	0.05	0.05	0.00	0.00	0.05	0.05	0.04	0.04
NR	0.08	0.08	0.06	0.06	0.07	0.06	0.00	0.00	0.06	0.07
NI	0.07	0.07	0.04	0.04	0.04	0.04	0.04	0.04	0.00	0.00

### Expected heterozygosity

Expected heterozygosity, as measure of variation within a pool, revealed that all pools exhibit similar levels of expected heterozygosity (Table 4) with RE and NI being highest and NR being lowest. When estimated for single pools, expected heterozygosity was between 0.271 and 0.283 for DA2 and DA3 and NR and between 0.280 and 0.285 in NI and RE. Estimated from the virtual union of both pools per colony, the values were about 0.01 higher, following the same trends (Table 5).

**Table 4:** Expected Heterozygosity within pools

	RE_1	RE_2	DA2_1	DA2_2	DA3_1	DA3_2	NR_1	NR_2	NI_1	NI_2
H_exp_	0.284	0.285	0.278	0.277	0.283	0.276	0.272	0.271	0.280	0.283
SD	0.188	0.185	0.189	0.189	0.189	0.190	0.193	0.193	0.189	0.185
Nloci [mio.]	16.497	16.497	16.496	16.496	16.496	16.497	16.497	16.497	16.497	16.498
NNA [mio.]	1′831	1′455	2′556	2′754	2′493	2′167	2′116	2′204	1′380	1′154

**Table 5:** Expected Heterozygosity estimated from the virtual union of both unit pools

	RE	DA2	DA3	NR	NI
H_exp_	0.298	0.292	0.294	0.285	0.295
SD	0.175	0.175	0.178	0.181	0.176
Nloci [M]	16.498	16.498	16.498	16.499	16.499
NNA	260	452	346	441	231

### Fixed alleles and private polymorphisms

Table 6 depicts the correlation of allele frequencies of loci that had complete recordings and where each colony was fixed for either the reference or the alternative allele (Supplementary Table 3). Only 506 loci fulfilled this criterion. The correlations between the colonies based on these loci ranged between − 0.10 and + 0.14, with the highest being between RE and NI.

**Table 6:** Correlation between genotypes for loci that were completely fixed within each unit

	RE	DA2	DA3	NR	NI
RE	1.00	−0.10	0.02	0.05	0.14
DA2	−0.10	1.00	−0.07	−0.02	0.01
DA3	0.02	−0.07	1.00	0.02	0.12
NR	0.05	−0.02	0.02	1.00	0.11
NI	0.14	0.01	0.12	0.11	1.00

On the other hand, RE held by far the largest number of still variable loci while the other pools were fixed at one allele. Out of the 1′203’000 loci fulfilling the criterion of being variable in one colony while all others were fixed, 555′591 belonged to RE (Table 7). NR (192′896) and NI (156′502) with about 80′000 loci carried more than the DA units (163′853 and 134′158).

**Table 7:** Number of private polymorphism; left: completely recorded loci; right: missing information (NA) allowed

	Without NA	with NA
RE	555′591	555′765
DA2	163′853	163′935
DA3	134′158	134′265
NR	192′896	192′974
NI	156′502	156′579

### Annotation

Functional annotation of loci significant in F-test, showing oppositely fixed alleles and exhibiting extreme F-test values revealed that most loci were in intergenic or intronic regions (compare Table 8, i.e. F-test: 65% intron and 19% intergenic) followed by ~ 13% upstream and downstream variants. Exonic variants were present to an extent of less than 1%. Potential protein changing variants like start or stop codons were barely present at a 5% significance level in the F-test and nearly absent among loci with oppositely fixed alleles. Compared to the unselected background, intergenic, intron, up– and downstream variants were slightly higher represented in both, the 5% F-test level and for the oppositely fixed loci, while exonic variants were in majority less frequent, especially for stop codons and in oppositely fixed loci only five such stop codons were present.

**Table 8:** Relative amount [%] of significantly differentiated and oppositely fixed loci per functional class and relative abundance of loci in differentiated classes in comparison to all background loci

	Relative amount of loci per class		Relative abundance compared to background	
	5% bonf	Opp. Fixed	5% bonf	Opp. fixed
3_prime_UTR_variant	1.0790	1.1905	0.9906	1.0913
5_prime_UTR_variant	0.2728	0.3345	0.9919	1.2232
coding_sequence_variant	0.0002	0.0000	0.9558	0.0000
downstream_gene_variant	6.4463	7.1600	1.0199	1.1224
intergenic_variant	19.9130	19.4910	0.9892	0.9698
intron_variant	65.4566	64.3371	1.0019	0.9848
missense_variant	0.2416	0.3683	1.0033	1.5229
start_lost	0.0009	0.0000	1.2111	0.0000
stop_gained	0.0020	0.0047	0.9747	2.1566
stop_gained,splice_region_variant	0.0001	0.0000	0.5296	0.0000
stop_gained,start_lost	0.0000	0.0000	5.2287	0.0000
stop_lost	0.0005	0.0000	1.0325	0.0000
synonymous_variant	0.5686	0.4842	1.0205	0.8975
upstream_gene_variant	6.0184	6.6297	0.9945	1.0989

**Fig. 5:**
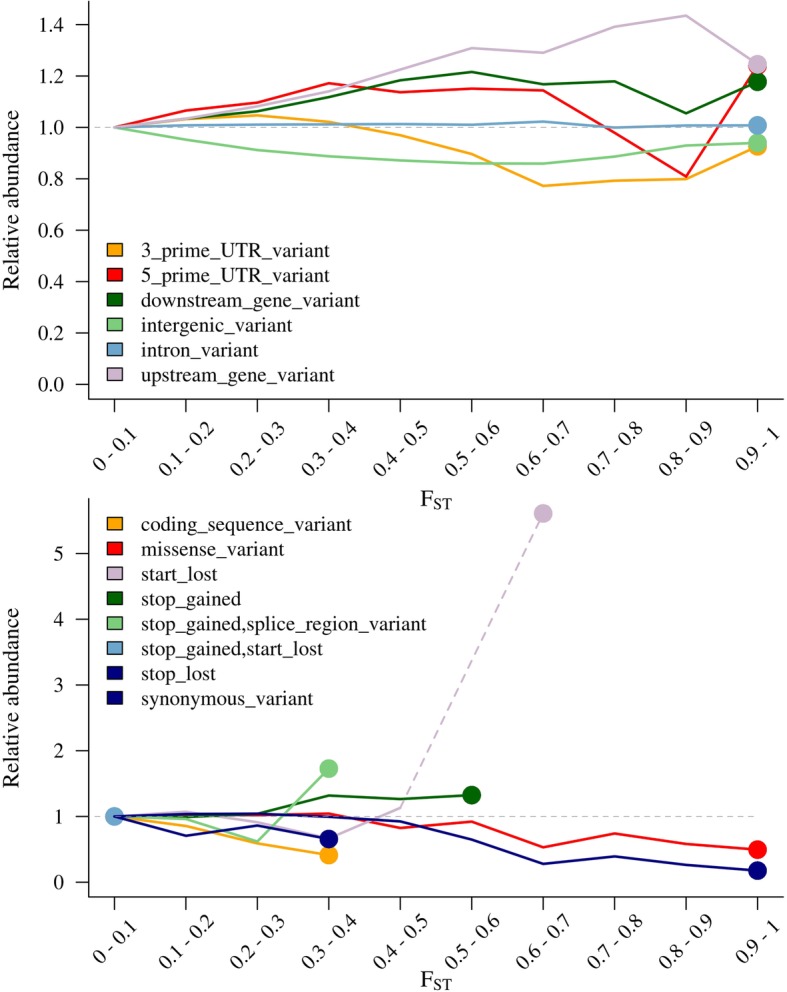
Relative abundance of functional SNP classes in dependence from pairwise FST bewteen units

Annotating SNPs in different levels of F_ST_ supported these findings. Start and stop codon changes could only be found at lower F_ST_ levels, while synonymous and missense mutations showed a decline in frequency towards high F_ST_ values, while up- and downstream, intron and intergenic variants were unaffected or increased in frequency (Fig. 5).

In the highest F_ST_ class with values > 0.9 there were 34 missense variants in all pairwise comparisons (Supplementary Table 4), 10 of them had a predictedly deleterious function (Table 9). While some annotations pointed to artefacts or novel genes, 3 of them were located in genes known by name, among them *Carbohydrate Sulfotransferase 12* (*CHST12*, DA2 vs DA3 and DA2 vs NI), *Arrestin Beta 1* (*ARRB1*, DA2 vs NR and NR vs. RE), Autoimmune Regulator (AIRE; DA2 vs. NR) and *Calcium/Calmodulin Dependent Protein Kinase II Beta* (*CAMK2B,* DA3 vs NR). RVIS scores for those genes were not extreme, with − 0.84 (among 11.18% most intolerant genes) *ARRB1*, − 0.62 (17.16%) for *CAMK2B*, − 0.57 (19.01%) for AIRE and 0.27 (70.58%) for CHST12.

**Table 9:** Annotation of deleterious missense variants with pairwise FST of 1

Chr	Pos [bp]	Pool 1	Pool 2	Ensembl ID	RS ID	SIFT	Gene name
3	1′741‘574	DA2	DA3	ENSSSCG00000007567	rs1108271931	0	CHST12
3	1′741‘574	DA2	NI	ENSSSCG00000007567	rs1108271931	0	CHST12
9	9′545‘629	DA3	NR	ENSSSCG00000014852	rs793754709	0	ARRB1
9	9′545‘629	NR	RE	ENSSSCG00000014852	rs793754709	0	ARRB1
10	2′699‘680	DA2	DA3	ENSSSCG00000042899	–	0.03	–
10	2′699‘680	DA2	NR	ENSSSCG00000042899	–	0.03	–
13	207′155‘372	DA2	NR	ENSSSCG00000025294	rs343158168	0.03	AIRE
14	132′394‘490	DA2	DA3	ENSSSCG00000046807	–	0.04	–
14	132′394‘490	DA2	NR	ENSSSCG00000046807	–	0.04	–
18	50′955‘382	DA3	NR	ENSSSCG00000039535	rs713007753	0.01	CAMK2B

Annotation of loci with variability in only one colony, while they were fixed in all other colonies, resembled the fractions of functional classes already known from the F-test and F_ST_ annotations (Table 10), but due to the higher number of private polymorphic loci in RE, the absolute numbers of loci annotated to potentially protein changing classes, such as missense mutations, was therefore higher in RE (2′380) than in all other colonies. DA3 colony carried the lowest number of missense mutations (538). Still, every colony carried at least one stop codon gain or loss, RE even 15.

**Table 10:** Relative amount (in per cent) of private polymorphism loci per functional class (absolute number of loci in brackets)

	RE	DA2	DA3	NR	NI
3_prime_UTR_variant	1.2176 (6317)	1.1844 (1790)	1.2121 (1516)	1.2375 (2211)	1.1192 (1634)
5_prime_UTR_variant	0.378 (1961)	0.3474 (525)	0.3622 (453)	0.4125 (737)	0.3199 (467)
coding_sequence_variant	0.0002 (1)	NA (NA)	NA (NA)	0.0006 (1)	NA (NA)
downstream_gene_variant	7.346 (38113)	7.1572 (10817)	7.1015 (8882)	7.2189 (12898)	6.6622 (9727)
intergenic_variant	39.5045 (204959)	39.6937 (59991)	41.8575 (52352)	39.7633 (71045)	40.8015 (59571)
intron_variant	43.3518 (224920)	43.5875 (65876)	41.7496 (52217)	43.179 (77148)	43.6521 (63733)
missense_variant	0.4587 (2380)	0.4268 (645)	0.4302 (538)	0.525 (938)	0.389 (568)
start_lost	0.001 (5)	0.0013 (2)	NA (NA)	NA (NA)	0.0007 (1)
stop_gained	0.0021 (11)	0.0053 (8)	0.0048 (6)	0.0022 (4)	0.0048 (7)
stop_gained,splice_region_variant	NA (NA)	NA (NA)	NA (NA)	NA (NA)	NA (NA)
stop_gained,start_lost	NA (NA)	NA (NA)	NA (NA)	NA (NA)	NA (NA)
stop_lost	0.0008 (4)	0.0007 (1)	NA (NA)	0.0011 (2)	NA (NA)
synonymous_variant	0.6557 (3402)	0.6213 (939)	0.5525 (691)	0.6571 (1174)	0.5082 (742)
upstream_gene_variant	7.0837 (36752)	6.9746 (10541)	6.7297 (8417)	7.0029 (12512)	6.5424 (9552)

## Discussion

The aim of our study was to determine whether the integrity of the breed Göttingen Minipigs was compromised by the current production and the genetic management system that relies on genetic isolation of production units. First, the classification of the GMP samples within the context of various pig breeds representing worldwide porcine genetic variation was evaluated with phylogenetic and population genetic methods. Secondly, genetic identity of the breed was assessed by multiple approaches describing variability within and differentiation between the separated barrier colonies.

### Discriminability of Göttingen Minipigs from other pig breeds

Our PCA, genetic distance and phylogenetic results show clearly distinct groups of European pigs, Asian pigs and Göttingen Minipigs. The distance between the European and Asian breeds reflects the current scientific consensus that domestication happened independently in Europe and Asia about 9000 years ago [8]. The European breeds appear generally closer to each other, which might be explained through the different domestication processes in both centers: while the European breeds emerged more or less directly from relatively uniform wild boar strains [9], the Asian domestication history is characterized by complex human driven dispersal of domesticated pigs in the South East Asian archipelagos, sometimes interrupted by feral states, before pigs eventually reached the Asian mainland [10]. This might explain why the European group clusters closely together in the UPGMA tree with higher resampling support than the Asian group. The tree, based on genome wide SNPs, clusters together, on one hand Xiang, Meishan and the South Chinese wild boars and on the other hand Jiangquhai and the North Chinese wild boars, interrupted by the Mini-LEWE. This is in contradiction to Ai et al. [11] who found that Meishan clustered together with the North Chinese wild boars, and could also support the low resampling probabilities found among the Asian breeds. The Mini-LEWE, a composite miniature breed, developed by crossing Vietnamese Potbellied Pigs, Saddlebacks and German Landrace, is in our study represented by a DNA pool of 10 females and a virtual pool made up from two sequenced individuals. Although it appears that individual sequences are not fully comparable to pools, since mixing of individual sequences and pool sequences leads to clustering of the respective sample types (results not shown). Still, the virtual and the DNA Mini-LEWE pools are clearly identified as one breed. Therefore, the virtual pooling seems to be a suitable measure to make different types of data comparable. In the case of the GMP, both types were mixed and both analyses, PCA and the UPGMA tree show that it is easily discriminable from all other breeds. The phylogenetic tree supports a GMP clade with 100% resampling support, which is located among the Asian pig breeds. This can be explained by the cross-breeding history in which Vietnamese Potbellied Pigs, Minnesota Minipigs and German Landrace were involved [12]. An earlier study [7] estimated that about 70% of the GMP genome are of Asian origin. In the PCA, the first component explains the difference between the three breed groups as the main source of variation, accounting for 19% of the genetic variability, while the second component discriminates the GMP and European breeds from the Asian breeds. Following the interpretation of Kim et al. [13], the average F_ST_ between the three groups, ranging between 0.26 and 0.33, suggests that still a major part of the total variability can be assigned to differences among individuals. Anyway, albeit using microsatellite data, they encountered similar estimates for F_ST_ values in a set of breeds comparable to this study. Therefore, we conclude that the GMP is still a distinct breed that can be easily distinguished from other breeds.

### Variation and differentiation within and between the GMP pools

While it seems particularly easy to distinguish the GMP from other pig breeds, it is more difficult, but relevant from a breeders’ point of view, to determine, if genetic isolation of the five breeding colonies has led to differentiated subpopulations. Applying PCA on the 10 GMP pools, we were able to see a trend to three subgroups consisting of NR, RE, and a cluster comprising NI and DA, respectively. The presence of a certain level of stratification is expected and has been observed beforehand, i.e. in studies in dogs [14] or sheep [15]. In the latter study, several breeds with heterogeneous breeding background split into sub clusters, for example dependent on their origin (American Suffolk vs British Suffolk, F_ST_ ~ 0.058–0.064; African vs American Dorpers, F_ST_ = 0.053) or phenotypic differences (Australian Poll Dorset vs American Dorset, F_ST_ = 0.082), while New Zealand and American Texel appeared indistinguishable (F_ST_ = 0.025). Studies comparing clearly distinct pig and cattle breeds, respectively, found F_ST_ values between 0.06 and 0.40 [16, 17]. F_ST_ of ~ 0.1 was found between relatively similar breeds, for example Large White and Landrace, while values higher 0.3 indicated major differentiation, such as between Nellore and Holstein cattle or Asian and European pig breeds. These values matched our findings between the European, Asian and GMP groups. Within the GMP, even two randomly composed pools from the same unit had a minimum differentiation of about 0.05. Between the aforementioned clusters F_ST_ was about 0.06 to 0.8 and therefore between the differentiation observed between the sheep breeds from separate origins and clearly distinct breeds. We explain this by genetic drift and slight differences in the actual breeding management, since the three clusters are confounded with the three partners in GMP breeding, even though all follow the same general breeding goal. We also assume that ascertainment bias affecting the variant discovery procedure might have an elevating effect on differentiation since incorporation of various pig breeds in the discovery sample might allow for calling more variants and especially more heterogeneous variants than calling in a GMP only sample [18].

Comparison of our results with the F_ST_ levels found in the aforementioned studies implies that our colonies are at the edge of splitting into sub-populations, and we did not expect all genomic regions between all pairwise combinations of the five units being similarly differentiated, when focusing on individual loci. The F-test (Table 3) identified about 4 to 8% of the genome to be objected by differentiation which is similar to the range found in a comparable study by Amaral et al. [19]. We hypothesize that genetic differentiation should be attributed to drift rather than to selection, if it affects neutral loci relatively more than loci with putative harmful consequences on protein translation, such as stop codon gains or deleterious missense mutations. This was supported by an underrepresentation of detrimental variation among highly differentiated loci. The 10 loci representing deleterious missense mutations with maximum F_ST_ were partly located in genes with known function. Although the RVIS values from the genetic intolerance analysis suggest that the respective genes are relatively tolerant to changes in functional variation, there should be further research on the real functionality, since some genes may have influence on important features of GMPs. While high differentiation in one candidate, *CAMK2B*, seems even vital to maintain cellular functions [20, 21], for example, *ARRB1* has found to be involved in feed conversion in pigs [22], *AIRE* is an important pathway gene of auto-immune response [23], and *CHST12* is differentially expressed in Oocytes after nuclear transfer in mice [24]. Nine of these SNPs are identified when the DA2 and DA3 subpopulation are involved in a pairwise comparison, three of them are between DA2 and 3 and five are between a DA unit and NR, indicating that there is detectable differentiation between the DA units and NR. When focusing on the subset of loci where all units are fixed for either allele, it is interesting, that the correlations between all units at such loci range around zero, indicating no clear pattern of shared fixed loci among colonies.

When we looked at expected heterozygosity as a measure of variability, RE and NI exhibit just slightly higher values than the other colonies, but it is notable, that RE holds about as many private polymorphisms as all other units together, making it an indispensable resource of genetic variability. We explain this with the consequent implementation of the mating scheme based on the optimum genetic contribution concept [25] in RE.

Not only is the preservation of a common genetic identity for all colonies of the GMP important [26], but also the risk of inbreeding depression and loss of variation due to drift is increased in artificially reduced subpopulations [6]. To counter this in future, two strategies appear feasible: First, the exchange of genetic material, e.g. via artificial insemination, and second, selection of a most diverse set of breeders as basis for future breeding. The first strategy, in which semen from RE, the major reservoir of remaining variability, would be used to inseminate breeders in the other units, as it is commonly used by dog breeders, would harbor various risks of spreading diseases and disorders between units. The second option of selecting a most diverse set of breeders from the respective unit also has the potential to increase heterozygosity, as can be observed in NI whose founding population was established in that very way. It can be taken as an example of the Bulmer effect [27] that genetic variation in the relatively large colony of DA3 wasn’t lost while selection and assortative mating was conducted, and could be largely recovered when the NI founders were chosen for maximum diversity.

## Conclusions

Our study based on assessment of differentiation found that the GMP as a breed is easily discriminable from other pig breeds. The finding of genetic distances and differentiation between the isolated breeding colonies of the GMP being minor in comparison to such between distinct breeds is taken as evidence of a successful conservation breeding program, even though some indications of stratification were detected. Functional annotation revealed that loci with functional impact are less differentiated than neutral loci, which implies that the force differentiating the colonies appears to be rather random drift than selective pressure. Selection pressures, through the current breeding activities, might on the other hand have ensured similarity of all colonies in regions which are expected to underlie traits important for the use of the GMPs as animal models. The detection of putatively deleterious highly differentiated variations in candidate genes with functions important for GMPs suggest that research needs to be undertaken to confirm their functionality in our animals.

Albeit animal exchange seems not yet necessary, the RE subpopulation harbors the highest amount of genetic variation while not being especially differentiated from all other colonies.

## Methods

### Samples

A joint pedigree was created from the pedigrees of all colonies in the five separated barrier facilities (Research Farm Relliehausen: Relliehausen (RE); Ellegaard Göttingen Minipigs A/S: Dalmose barrier 2 and 3 (DA2, DA3); Marshall BioResources: North Rose (NR); Oriental Yeast Co., Ltd.: Nisshin (NI)). Numerator relationship matrices were constructed with Wrights coefficient of relationship [28] for each colony and all animals alive within a colony in November 2015. A set of 30 individuals was selected for blood sampling with the following procedure in each colony, respectively: all candidates available for blood sampling consisting of only non-pregnant, healthy sows without genetic disorders were identified. A subset of 30 animals was randomly sampled from this list and the relationship within the set (*a*) and between the animals in the set and all remaining animals in the colony (*b*) were calculated. Both values were combined in an index *I* = 0.8*a − 0.2*b, to minimize relationship within the samples while maximizing relationship with the sample and the remaining colony. This sampling was repeated up to 25′000 times and restarted every time a new index value went below the previously recorded one. The procedure was stopped after 25′000 rounds without improvement.

DNA of two times ten animals per colony, randomly chosen from the available samples from the previously selected 30 candidates, was pooled using equimolar amounts of the individual DNA. 150 bp paired-end sequencing was done on an Illumina HiSeq 4000 with an aim coverage of 30X and an insertsize of about 420 bp. Publicly available data from 13 various pig breeds [29–31] as used in a previous study [32] were incorporated (see also Table 11). Raw data was aligned to the reference genome Sscrofa11.1 [33] with BWA 0.7.12 [34], sorting, merging of different libraries and marking duplicates were done with Picard tools 2.10.5 [35], base qualities were recalibrated with GATKs BQSR [36, 37] using the available SNPs from dbSNP as validation [38]. Biallelic SNPs were called with the Haplotype Caller from GATK 4.0.8.1 in gVCF-mode. SNPs were filtered with the VQSR tool of GATK that uses machine learning to assess the validity of a SNP. SNPs contained in the Affymetrix Axiom_PigHD_v1 array were used to train the model incorporating the variant attributes QualitybyDepth (QD), MappingQuality (MQ), MQRankSumTest, ReadPositionRankSumTest, FisherStrand (FS), StrandOddsRatio (SOR) and depth (DP). A truth sensitivity filter level of 99.0 was applied. Monomorphic loci, X-chromosomal loci and loci without records were removed.

**Table 11:** Additional porcine samples used in Reimer et al. (2014)

Breed	Number of Samples	Average Depth	Class	Subclass
Duroc	4	5.98	European	Domestic
Hampshire	2	6.49	European	Domestic
Jiangquhai	1	8.20	Asian	Domestic
Large White	14	6.46	European	Domestic
Landrace	5	6.36	European	Domestic
Meishan	4	6.83	Asian	Domestic
Pietrain	5	5.61	European	Domestic
Xiang	2	6.27	Asian	Domestic
European wild boar	6	6.44	European	Wild
Asian wild boar	5	6.27	Asian	Wild
Göttingen Minipigs external	1	12.76	Minipig	Göttingen
Göttingen Minipigs	10	13.01	Minipig	Göttingen
Mini-LEWE	2	13.93	Minipig	Berlin
Mini-LEWE pool	10	13.14	Minipig	Berlin
Wuzhishan	1	11.02	Asian	Domestic

### Principal component analysis (PCA)

PCA was constructed from matrix ***X***, which contained samples in rows and genetic loci in columns. Elements of ***X*** were the respective reference allele frequencies. Every locus was centered and scaled beforehand. Due to the nature of data from short-read sequencing, matrix ***X*** contained numerous missing values. To account for these, missing information was recoded in matrix ***N*** with the same dimensions as ***X***, in which records and missing positions were recoded as 1 or 0, respectively. The adjusted covariance structure was subsequently modeled as $\boldsymbol{E}=\frac{\boldsymbol{XX}\hbox{'}}{\boldsymbol{NN}\hbox{'}}$. Eigenvectors ***V*** and eigenvalues **λ** were achieved bei eigenvalue decomposition of ***E.*** Loadings ***L*** where the corrected eigenvalues where negative values were set to zero. Principal components were calculated by multiplying the eigenvectors with a diagonal-matrix containing square roots of ***L***.

### Fixation index and Reynolds distance

Fixation index (F_ST_) and Reynolds distance (D_R_) were estimated between breed pools. Therefore read information of individuals was virtually pooled by breed-wise summation of reads supporting the reference and the alternative allele, respectively.

Reference allele frequency in each breed *k* per locus was estimated as ${p}_k=\frac{R_{ref}}{R_{ref}+{R}_{alt}}$, with *R*_*ref*/*alt*_ denoting the number of reads supporting either the reference or alternative allele, and *F*_*ST*_ calculated per locus as ${F}_{ST}=\frac{H_T-{\overline{H}}_S}{H_T}=\frac{\overline{p}\ast \left(1-\overline{p}\right)-\frac{p_1\ast \left(1-{p}_1\right)+{p}_2\ast \left(1-{p}_2\right)}{2}}{\overline{p}\ast \left(1-\overline{p}\right)}$, with $\overline{p}=\frac{p_1+{p}_2}{2}$. Reynolds distance was estimated as ${D}_R=\frac{1}{2}\ast \frac{\sum \limits_{i=1}^2\left({p}_{1i}-{p}_{2i}\right)2}{1-\sum \limits_{i=1}^2{p}_{1i}{p}_{p2i}}$, where *i* reflects the *i*^*th*^ allele at a biallelic locus, namely the reference allele or the alternative allele, respectively [39]. Both measures were averaged over all pairwise complete loci to gain genome-wide values

### Phylogeny

A phylogenetic tree was constructed from genome-wide *F*_*ST*_ values from all autosomal loci, using the clustering algorithm UPGMA as implemented in the package “phangorn” [40]. The resulting tree reliability was determined by comparison to 100 trees constructed from 100 randomly sampled loci each.

### Test of allele frequency differences between pools

We employed an F-test based statistic to determine statistically significant variation patterns between pools for every locus (eq. 1).

1 $$ F=\frac{V_I}{V_O}, $$

where *V*_*I*_ is the pooled variance within a unit, e.g. RE1 and RE2, estimated as ${V}_I=\frac{p_{RE1}+{p}_{RE2}}{2}\ast \left(1-\frac{p_{RE1}+{p}_{RE2}}{2}\right)\ast \frac{2}{10}$, and where *V*_*O*_ represents the variance between the aforementioned unit and a remote pool, e.g. NI1 estimated as $\frac{p_{RE1}+{p}_{NI1}}{2}\ast \left(1-\frac{p_{RE1}+{p}_{NI1}}{2}\right)\ast \frac{2}{10}$. The degrees of freedom where assumed to be nine, since every pool was made up from ten animals.

### Heterozygosity, fixed alleles and private polymorphisms

Expected heterozygosity at locus *i* was estimated from original pools and the virtual pool for each colony as ${H}_{{\mathit{\exp}}_i}=2\ast {p}_i\ast \left(1-{p}_i\right)$, where *p*_*i*_ is the reference allele frequency. It was further assessed whether a single colony was fixed for one allele, while the others were fixed for the other allele. To assess variability remaining in only one colony, loci where all colonies apart from one were fixed were identified. This was done both for the subset of loci without missing information and for loci where single colonies had missing information.

### Annotation

Loci identified in the aforementioned tests were functionally annotated with the Ensemble Genes database (version 98; Sscrofa11.1 [41]). Genetic intolerance of predicted deleterious missense mutations at highly differentiated loci was assessed by calculation of Residual Variation Intolerance Scores (RVIS [42]).

## Supplementary information

**Additional file 1 Supplementary Table 1**. Genome wide FST (upper triangle) and Reynolds distance (lower triangle).

**Additional file 2 Supplementary Table 2**. Number of SNPs significant at 5% Bonferroni corrected significance level in F-Test.

**Additional file 3 Supplementary Table 3**. Functional annotation of SNPs oppositely fixed between one unit and a distant pool from another unit.

**Additional file 4 Supplementary Table 4**. Functional annotation of SNPs dependent on the F_ST_ value between two colonies.

## Data Availability

Short read data of the DNA pools is available in the European Nucleotide Archive (ENA) under project accession PRJEB36673. Public data used in this study can be found in ENA: ENA accessions for FASTQ files of samples from Rubin et al. [29]: ERR173170, ERR173171, ERR173172, ERR173173, ERR173174, ERR173175, ERR173179, ERR173180, ERR173181, ERR173182, ERR173183, ERR173184, ERR173185, ERR173186, ERR173187, ERR173188, ERR173189, ERR173190, ERR173191, ERR173192, ERR173193, ERR173194, ERR173195, ERR173196, ERR173197, ERR173198, ERR173199, ERR173200, ERR173201, ERR173202, ERR173204, ERR173205, ERR173206, ERR173207, ERR173208, ERR173212, ERR173213, ERR173214, ERR173215, ERR173216, ERR173217, ERR173218, ERR173219, ERR173220, ERR173221, ERR173222, ERR173223, ERR173224; Wuzhishan Samples from Fang et al. [30]: SRR448575, SRR448588, SRR448589, SRR448591, inititially accessed through ftp://climb.genomics.cn/pub/10.5524/100001_101000/100031/reads/; SRA accessions for GMP samples from Vamathevan et al. [31]: SRR578029, SRR578191, SRR578192; ENA accessions for the GMP and Mini-LEWE data [32]: PRJEB27654.

## Abbreviations

DA: Colony of Dalmose, Denmark
DNA: Deoxyribonucleic acid
D_R_: Reynolds genetic distance
F_ST_: Fixation index
GMP: Göttingen Minipigs
KEGG: Kyoto Encyclopedia of Genes and Genomes
M: Million
Mini-LEWE: Minischwein Lehnitz-Wendefeld
NI: Colony of Nisshin, Japan
NR: Colony of North Rose, NY, USA
PCA: Principal component analysis
RE: Colony of Relliehausen, Germany
SNP: Single nucleotide polymorphism
UPGMA: Unweighted pair group method with arithmetic mean
